# A New Methodology to Assess Fallopian Tubes Microbiota and Its Impact on Female Fertility

**DOI:** 10.3390/diagnostics12061375

**Published:** 2022-06-02

**Authors:** Salvatore Giovanni Vitale, Jose Carugno, Maurizio Nicola D’Alterio, Mislav Mikuš, Pasquale Patrizio, Stefano Angioni

**Affiliations:** 1 Obstetrics and Gynecology Unit, Department of General Surgery and Medical Surgical Specialties, University of Catania, 95124 Catania, Italy; 2 Minimally Invasive Gynecology Unit, Obstetrics, Gynecology and Reproductive Sciences Department, Miller School of Medicine, University of Miami, Miami, FL 33136, USA; tonycarugno@yahoo.com (J.C.); pxp612@med.miami.edu (P.P.); 3 Division of Gynecology and Obstetrics, Department of Surgical Sciences, University of Cagliari, 09042 Cagliari, Italy; mauridalte84@gmail.com (M.N.D.); sangioni@yahoo.it (S.A.); 4 Department of Obstetrics and Gynecology, University Hospital Center Zagreb, 10000 Zagreb, Croatia; m.mikus19@gmail.com

**Keywords:** female infertility, hysteroscopy, laparoscopy, pelvic inflammatory disease (PID), tubal microbiota

## Abstract

Tubal factor is an important contributor to female infertility, and the current diagnostic approaches cannot correctly identify many subtle causes of tubal dysfunction. While it is known that the most common cause of tubal factor infertility is pelvic inflammatory disease (PID), creating critical alterations of the tubal epithelium, little attention has been devoted to understanding the tubal modifications caused by the resident microbial population and their interaction with the surrounding tubal epithelium. Furthermore, most of these samples are obtained by traumatic procedures such as direct sampling during laparoscopy using a cytobrush. However, as in any other organ of the female genital tract, the microbiota environment of the fallopian tube plays an essential role in maintaining tubal functioning, counteracting the pathogenic effect of acquired microbes. Consequentially, to better analyze the tubal microbiota without causing anatomical and/or functional alteration of the fallopian tube and preserving fertility, the hysteroscopic approach might be the method of choice, guarantying maximal integrity of the uterine cavity and tubal lumen. Here we describe our plan for using atraumatic hysteroscopic sampling methods to investigate the correlation between tubal microbiota and female infertility.

Tubal factor causes 30% of all female infertility and generally occurs secondary to tubal obstruction, distortion, or intrinsic dysfunction of the epithelium [1]. The most prevalent cause of tubal factor infertility is pelvic inflammatory disease (PID) which is caused by several pathogens. PID affects tubal patency not only with macroscopic structural distortions [2] but also by affecting the tubal epithelium directly [3,4]. Various cytological studies demonstrated that several PID pathogens (*Chlamydia trachomatis*, *Neisseria gonorrhoeae*, *Escherichia coli*, *Mycoplasma hominis*, *Mobiluncus*, *Bacteroides* and *Ureolyticus*) cause ultrastructural changes consisting of sloughing and/or destroying of ciliated cells, with subsequent cessation of ciliary activity, disruption of cell junctions and apoptosis of epithelial cells. These are the consequences of several pathogenic mechanisms such as direct cytotoxic effect, immune response, secretion of chemokines, and cytokines [5,6].

Several studies have confirmed that each organ of the female genital tract is characterized by the presence of a specific microbial population that plays a pivotal role in maintaining the normal reproductive function, and preventing dismicrobisms and infections [7,8].

Similar to what happens in the vagina and the endometrium, it could be argued that the microbiota, as a result of a delicate balance of the all-female genital tract organs, also plays a prominent role in the tubal function and, therefore, in female fertility opposing to pathogenic microbes. With this in mind, a few authors have focused their efforts on the direct evaluation of microbe’s effect on the tubal lumen, assessing, incidentally, only the detection of the most common pathogens.

The majority of data currently available in the literature come from studies obtained by laparoscopic access, specifically by salpingectomy [9]. Other samples were retrieved by direct biopsies of the distal portion of the fallopian tube during laparoscopic procedures [10]. It is important to consider that surgical excision of the Fallopian tubes represents a methodological bias for microbes and cytological sampling for several reasons. First, mechanical manipulation and electrocoagulation may impair the microbiological and cytological content. Second, the salpingectomy reduces the analysis area to a restricted portion of the fallopian tube without considering the microbes present in the entire tubal lumen. Furthermore, tubal microbiota results from a dynamic steadiness derived from the anatomical and functional integrity of the whole internal female genital tract. Thus salpingectomy, interrupting this integrity, might interfere with this delicate balance.

To maintain the anatomical and functional integrity of the whole female genital tract and obtain a cytological and microbiological sample of the tubal epithelium, finding a non-invasive technique becomes mandatory. In this perspective, the hysteroscopic approach could be the method of choice for an indirect tubal integrity evaluation (cytological and microbiological). Currently, hysteroscopy is considered the ideal diagnostic procedure for assessing the vaginal walls, cervical canal, uterine cavity, endometrium, and tubal ostia [11,12,13]. Moreover, hysteroscopy could be performed in an office setting without the need for anaesthesia, using modern miniaturized hysteroscopes equipped with a 5 French operative channel, ensuring the direct vision of the structures without compromising their integrity [14].

Some authors have used the hysteroscope to obtain fallopian tissue samples to determine the etiologic diagnosis of salpingitis using a cytobrush inserted through the working channel [15]. The hysteroscopic approach seems more appropriate than the laparoscopic approach because of its less invasiveness and lower cost. However, the use of cytobrush has some critical implications worth highlighting. First, the unavoidable mechanical trauma caused by the cytobrush may irreversibly damage the fallopian tube epithelium, causing iatrogenic tubal lesions and potentially impacting future fertility. Furthermore, the diameter and the limited flexibility of the cytobrush make this device inadequate for the microbiological and cytological sampling of the distal tubal lumen.

Although hysteroscopy seems the most appropriate minimally invasive method to examine the tubal factor, scanty and inconsistent data are currently available in the literature to support its use.

In conclusion, the tubal infertility factors remain an enigmatic dilemma waiting to be discovered entirely, and the microbiota, with its changes, could be a milestone in the understanding of tubal factor infertility. Although vaginal and endometrial microbiota are extensively studied in the literature [7,8], scant evidence can be gathered regarding tubal microbiota [16]. Consequentially, understanding the tubal microbiota in relation to some demographic variables such as ethnicity, parity, and history of infertility could be relevant to understanding the role of these microbes in maintaining the tubal patency.

For this purpose, alternative microbiological and cytological sampling methods should be encouraged without the potential methodological bias described above. Thus, large multicentre well-designed studies using hysteroscopic sampling methods are necessary to elucidate the relationship between tubal microbiota and female infertility.

## Data Availability

Not applicable.
